# Driving through dense fog: a study of the effects and control of sustainable public procurement of electric cars

**DOI:** 10.1007/s10669-022-09854-2

**Published:** 2022-04-21

**Authors:** Marius Langseth, Helene Tronstad Moe

**Affiliations:** 1 Kristiania University College, 0186 Oslo, Norway; 2 The Norwegian University of Science and Technology, PB 1190, 7091 Trondheim, Norway; 3 The Arctic University of Norway, 9019 Tromsø, Norway

**Keywords:** Public procurement effects, Electric cars, Data-driven decisions, Sustainability

## Abstract

Governments are large buyers of vehicles, thus contributing to pollution. To promote sustainability, policies have been shaped to replace government-owned fossil fuel cars with electric cars. Public procurement is seen as a strategic tool for the government to transition. This study identifies a research gap due to a lack of studies on how stakeholders at different levels identify and calculate the sustainability effects of public procurement of cars. Our approach uses a multilevel perspective to explore how various stakeholders perceive and assess the effects of sustainable public procurement. The data were obtained through a qualitative research design with documents and semi-structured interviews with stakeholders in Norway ranging from government agencies, public procurement officers, car suppliers, and end-users. (End-users in this setting are the ones who ultimately use the vehicles). The study’s findings are two-fold. First, it contributes to understanding that perceived effects of sustainable public procurement vary from the stakeholders' perspectives and that public procurement initiatives perceive to have cultural effects in addition to innovation, environmental, economic, and social impacts. Second, it contributes to understanding the importance of feedback mechanisms in public procurement to align the assessment of the effects. A better understanding of how effects are identified, and improved feedback mechanisms could help government representatives control the procurement system and accomplish the intended effects.

## Introduction

While a well-functioning transport system is a prerequisite for achieving many of society’s objectives, emissions from car transport must be drastically reduced to meet the Paris Agreement (2015) goals. In 2017, road transport accounted for 21% of the EU's total carbon dioxide emissions (2021) and 17% of the emissions in Norway (NEA, 2020). One suggested intervention is to replace fossil fuel cars with Electric Vehicles (EVs). To increase the number of EVs, Norway has introduced some of the most effective incentive schemes globally (Aasness and Odeck, 2015). Some of the incentives are that EVs are exempt from value-added tax, cheaper road tax, and parking. The incentive schemes have contributed to a change in buyers’ behaviour, and according to numbers by the Norwegian Road Federation (OFV, 2022), in 2021, 64.5% of new cars sold were electric. A large spender and owner of cars is the government itself. In Norway, the state, counties, and municipalities buy goods and services related to transport, amounting to approximately 1 billion EUR annually (Meld. St. 22, 2018–2019).

In this context, public procurement is often highlighted as a strategic instrument to stimulate sustainable consumption and cleaner production. Sustainable public procurement is high on the agenda of European policymakers, for example, reflected in the EU’s ‘Fit for 55’ (2021) and the Norwegian government’s climate plan 2021–2030 (Meld. St. 13, 2020). The climate plan argues that public procurement can play an essential role in reducing CO2 output and stimulating innovative green solutions. One action point is replacing fossil fuel cars with EVs by making it mandatory from 2022 for the public sector to buy EVs. The UN Sustainable Development Goals (SDGs) also reflect public procurement’s sustainability potential, where SDG target 12.7 (2015) is connected to public procurement. An OECD assessment of the public procurement system in Norway showed that Norway had a solid legal and regulatory foundation for sustainable public procurement, which, however, was not well-implemented (OECD MAPS, 2020). The findings were also confirmed by a survey conducted by The Norwegian Agency for Public and Financial Management (2020), where the results showed that although 60% of the public procurement entities had an environmental procurement policy, lack of time, competence, and monitoring systems were the most significant barriers against implementation.

Earlier research has studied how procurement can drive the circular economy, e.g. (Alhola et al. 2019), the procurement of sustainable innovation, e.g. (Rolfstam 2015), and public procurement as an environmental policy mechanism, e.g. (Aldenius & Khan 2017). When looking at sustainability and effects, other studies have aimed to explore the relationships between environmental energy sustainability, low-carbon energy, and climate change mitigation, e.g. (Ionescu 2021a, b, c) and solve the practical problem on how to measure sustainability, e.g. (Neri et al. 2021). There is a research gap in studies on how the effects are perceived and assessed from different stakeholders’ perspectives. Based on the introduction, the paper aims to answer two research questions: RQ1: How do government agencies, procurement officers, car suppliers, and end-users perceive the effects of sustainable public procurement of cars? RQ2: How do these stakeholders assess the effects of sustainable public procurement of cars?

The study proceeds as follows. Section two extends the introduction and lays the foundation for further work. Section three presents the research design and provides an overview of data collection and analysis. Further, Section four evaluates the findings, and Section five discusses the implications of the results, thus presenting the research limitations and suggestions for further research. Finally, Section six concludes the study.

### Related research

Various theoretical frameworks have been used to investigate sustainability effects in general and public procurement of cars more specific. One of the first and widely used frameworks for exhibiting effects is The Triple Bottom Line (TBL), introduced by Elkington (1997). Elkington argues that organisations should see beyond the traditional economic bottom line and expand their reporting to include social and environmental factors. The TBL concept has since Elkington developed in many directions, and we will briefly go through the most influential ones. One of the frameworks that highlight sustainability effects is the Global Reporting Initiative (GRI). GRI is an international independent standard that aims to help organisations communicate their impact on climate change and human rights issues. According to KPMG (2017), 67% of the largest 100 companies in 2020 used GRI for reporting.

TBL has also given birth to terms like Corporate Social Responsibility (CSR) and Environmental, Social, and Governance reporting. Porter and Kramer (2011) expanded the concept of CSR and introduced the concept of Creating Shared Value (CSV). One of the ideas behind CSV is that the success of a company and its suppliers are mutually dependent. Integrated Reporting, introduced by Gleeson-White (2015), is another framework influenced by TBL. In line with TBL, it emphasises that organisations should highlight the value it generates for society and the environment, but adds that this should be done along six capitals. In 2015, the United Nations presented the Sustainability Development Goals (SDG). These are a collection of 17 interlinked global goals to achieve a sustainable future; further, in 2017, the SDGs came with specific targets and indicators to measure progress. Countries report to the UN on their efforts, and Norway published a voluntary review in 2021 (VNR 2021). Lately, there has been discussion on whether the SDG goals are contradictory (Nilsen 2020), interlinked (Fonseca et al. 2020), or whether the environmental goals are a prerequisite for others (Singh et al. 2018). In a later article, Elkington (2018), who kick-started the reporting movement, proposes to recall the TBL framework. The main problem, he argues, is that organisations have smartly used the TBL to show how commendable they are. As Elkington (2018) explains, “Together with its subsequent variants, the TBL concept has been captured and diluted by accountants and reporting consultants”.

### Effects of public procurement of cars

Research connected to public procurement, sustainability, and cars has resulted in multifarious initiatives. Kemp and Rotmans (2004) suggest that transition into sustainable transport should be done in small steps. They call this transition management. Vergragt and Brown (2007) indicate a re-learning of society related to personal mobility, where the government plays a part in stimulating innovation. Michelsen and de Boer (2009) find that public procurement officers put sustainability demands in their calls for tenders. However, that lowest cost was often the actual selection criteria of the supplier. Whitmarsh and Köhler (2010) highlight the role of policy drivers in innovation in the supply and demand of cars and argue for greater attention to psychological, cultural, and infrastructural factors. Villareal (2011) describes what he calls an ‘imitative rationality’, wherein the market for EVs is a cognitive battle to define personal mobility. Brammer and Walker (2011) and Shepherd et al. (2012) show a wide variation in involvement in implementing sustainable procurement when there is a demand for cars. However, notably, if the senior managers were supportive, the procurement team would be more likely to implement changes. Tran et al. (2013) find that financial benefits, rather than pro-environmental behaviour, were the most significant influence on adopting environment-friendly solutions. van Rijnsoever et al. (2013) show that the local Dutch governments were willing to pay between 25 and 50% extra for an alternative fuel vehicle without a severe loss of utility.

Nykvist and Nilsson (2015) studied what they called the EV paradox in Sweden. They observed that despite favourable conditions, the adoption of EVs was low. They explained that this was due to a regime favouring hybrid plugin vehicles. Palm and Backman (2017) also studied EVs in Sweden. They found that charging infrastructures and costs were barriers to diffusion. Ydersbond (2018) studied municipalities in Norway and found that the primary reasons to adopt EVs were political signals, economic benefits, and entrepreneurial employees who worked to promote electric cars. Significant barriers to adoption included the need for four-wheel drive, driving range, and structural conditions such as the length of leasing contracts. Mulligan (2021) points out new opportunities in smart city developments and argues that the Internet of Things and data analytics are instrumental for automated algorithmic decision-making processes. Finally, both reviews by Patrucco et al. (2017) and Sönnichsen and Clement (2020) show that the general scientific literature on sustainable public procurement is broad and growing. Literature related to public procurement of EVs involves leadership involvement, innovation, and adoption. However, there is a need to look further at how the effects are perceived based on the interest of various levels of stakeholders.

### Calculations and assessment of procurement effects

Regarding the second research question on how the actors assess the effects, Thai (2001) argues that the feedback mechanisms are essential for a sound procurement system. Without a functioning feedback mechanism, it is difficult for policymakers and managers to see the consequences of their decisions. Van Thiel and Leeuw (2002) argue that the increase in performance assessment in the public sector could lead to lower performance because of a weak correlation between performance indicators and the performance itself. To counteract these consequences, they suggest multiple indicators reflecting the interests of different stakeholders and multidimensionality on various levels (micro, macro, and meso). Moe (2006) found that actors in the construction industry frame and calculate environmental-friendly houses differently.

Brynjolfsson et al. (2011) argue that organisations that use Data-Driven Decision Making show better performance. Sparrevik et al. (2018) studied the implementation of green public procurement in a building project. They found that data and co-creation between policymakers and regulators were critical for success. There is also existing literature that has been critical of the concept of sustainability measurement. For example, Boiral et al. (2020) show that sustainability performance is not a clear, measurable concept but an ambiguous phenomenon and that its rational appearance should be questioned. Lately, research has focussed on the relationship between advanced sustainability analytics, corporate social responsibility, and environmental sustainability. Keane (2020) explored the opportunities in self-driving cars. May et al. (2021) tested the inter-relationship between corporate social responsibility, employee green behaviour, and environmental sustainability. They argued that corporate social responsibility and employee green behaviour mediated by organisational trust and organisational identification positively affected environmental sustainability. The literature for adopting data-driven decision-making has explored the reasons for a mismatch between the simultaneous overproduction and underconsumption of data in government (Chen and Lee 2018). For instance, Langseth and Haddara (2021) found that even if public procurement officers used more digital tools and generated vast amounts of data, they were often unlikely to gather insights from these data and use it to make decisions.

The purpose of calculations in the sustainability field is to measure impact. Without calculations, representations cannot be designed or interpreted, and without measurement methods, one cannot estimate the extent to which criteria measure different sustainability aspects. Construction and management of representations, environmental criteria, and indicators require new calculations. The related research is summed up in the following table.

As presented, past studies have emphasised how leadership involvement and innovation are related to adoption and not distinguish the understanding of the effects of public procurement and EVs. The review shows various initiatives connected to assessing the impact, but these have concentrated on performance measurement and data-driven decisions. While studies in the past have studied discussed effects, there is a research gap in looking at the whole public procurement system and how the effects are perceived and assessed from different stakeholders’ perspectives. This study endeavours to address this limitation of previous research by looking at public procurement decision-making through the theoretical lens of management control activity where the various stakeholders represent the procurement system. In the related research, there is a research gap related to a lack of studies on the perceived effects of public procurement policy from different stakeholders’ perspectives. Table 1 summarises related research.

**Table 1:** Summary of related research

Effects of public procurement of cars			Calculations and assessment of procurement effects	
Leadership involvement	Innovation	Adoption	Performance management	Data-driven decisions
Brammer and Walker (2011) Shepard (2012) Sparrevik et al. (2018)	Edler et al. (2005) Whitmarsh and Köhler (2010)	Vergragt and Brown (2007) Michelsen and DeBoer (2009) Villareal (2011) Palm and Beckman (2017) Ydersbond (2018)	Van Thiel and Leeuw (2002) Eccles et al. (2014)	Brynjolfsson (2011) Boiral et al. (2020) Keane (2020) May et al. (2021) Langseth and Haddara (2021) Mulligan (2021)

### Public procurement decision-making as a management control activity

**Fig. 1:**
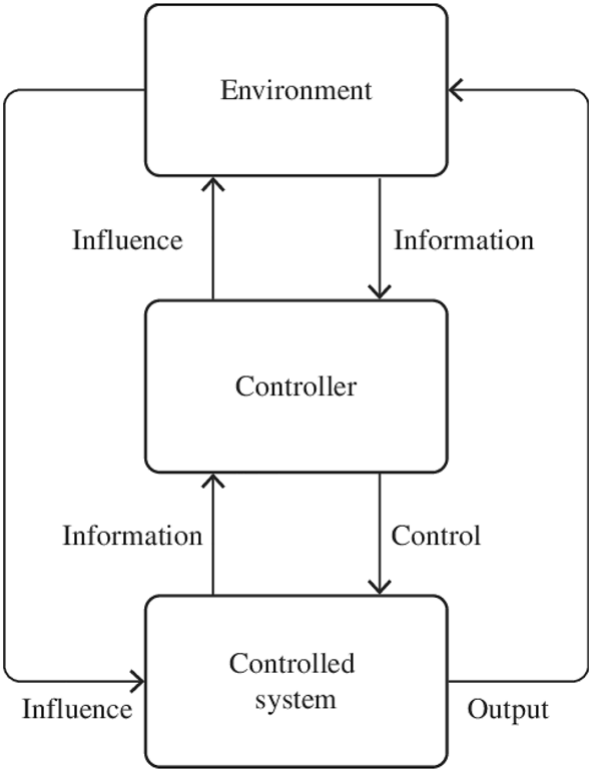
De Leeuw control paradigm (De Leeuw, 1976)

Schaltegger and Burritt (2010) describe sustainability assessment as a subset of accounting concerned with the methods, and systems used to assess and report economic, social, and environmental impacts and the relationships between these dimensions of sustainability. Sustainability assessment could then be considered a part of formal management control activities such as planning, evaluation, coordination, and procedure (Anthony et al. 2007). De Leeuw (1976) argues that management control activities should function as dual control relationships between an organisation and its environment (see Fig. 1). In his article, he argues that dual control belongs to the axiomatic branch of system theory. This branch defines the systems as a modelling box with abstract concepts and models filled with empirical content. The controller’s objectives are two-fold; (1) Action: To control the system based on current system knowledge, (2) Investigation: To experiment with the system to learn about its behaviour to control it in the future better. These two objectives may be partly in conflict. If you, for example, are driving an EV and want to be in control to get to your destination smoothly, you also want to experiment with how far you can go before a recharge.

According to De Leeuw, the controller’s ability to successfully control its system depends on the following five preconditions for adequate control.

1. The controller has an objective and an evaluation mechanism to check whether the goals are met.
2. The controller has a model of the controlled system to predict the effect of potential control actions.
3. The controller has information about the environment and the controlled system.
4. The controller has sufficient control actions to cope with the variability of the system.
5. The controller has sufficient information processing capacity to transform incoming information into practical control actions aligned with the objectives.

Central to De Leeuw’s control paradigm is information for the controller both from the environment and the controlled system to monitor and make better decisions. Weber (2011) suggests that, due to technological developments, the role of the controller will increasingly focus on decision support in the form of data analytics. Data analytics offers the controller the opportunity to elevate its role within an organisation, thus adding value to the business. This paper examines the public procurement system as an abstract control system that we want to fill with empirical content. By taking a system theory approach, we wanted to understand better the stakeholder's perspectives associated with public procurement in the context of EVs. We will use this perspective further when discussing our results. A description and overview of the research design, data collection methods, and data analysis follow.

## Methods and data

### Design

This study uses a grounded theory approach (Glaser and Strauss, 2017) for inductive theory-building to understand how stakeholders at different levels perceive and assess the effects of public procurement. An exploratory research design was chosen, and data were collected through a literature study, qualitative semi-structured interviews, and government documents. A qualitative method was selected to explore and understand how stakeholders perceive the sustainability effects. The qualitative approach is based on Eisenhardt (1989), who argues that the starting point for qualitative methods is that all phenomena comprise unique combinations of qualities that cannot be counted, measured, or weighed. We acknowledge qualitative research as a contextually situated practice.

Further, we recognise conceptualisation of qualitative research as research that is qualitative in both methods and values (Braun and Clarke 2013; Grant and Giddings 2002). As qualitative researchers, we understand contexts and qualitative researchers as contextually and temporally situated practitioners (Braun and Clarke 2021). Interview data are challenging to generalise but getting close to the informants gives us insight into the informant´s world of life (lebenswelt), which provides high validity. No qualitative research has much value without specific skills and practices as reflexivity, interpretation, and imagination (Gabriel 2018). Reflexivity can be understood as an “interpretation of interpretation” (Alvesson and Sköldberg 2009) and the extent to which reflexivity can corroborate the validity and enhance the value and contribution of an interpretation (Gabriel 2018). The goal was to understand and communicate how different stakeholders from various levels perceive reality related to sustainability. The grounded theory approach allows researchers “to make statements about how actors interpret reality” (Suddaby 2006). Therefore, the most crucial factor in collecting the data was gathering these stakeholders’ perspectives. It is recommended that the sampling process in grounded theory studies should involve the recruitment of participants and organisations that are perceived as experts in the subject matter (Edmondson and McManus 2007; Makri and Neely 2021). Thus, in the research design, we invited experts in a government agency specialising in sustainable public procurement to suggest study participants and organisations.

### Data collection

The project was reported to the Norwegian Centre for Research Data (NSD) before the data collection due to the data use privacy policy. As preparation for the interviews, document analyses regarding relevant government documents were conducted. The literature study collected data through Google Scholar searches using the following keywords: ‘sustainable public procurement’ AND ‘public procurement’ AND ‘green’ AND ‘car transport’, AND ‘effects’. A total of 17 relevant contributions were found and categorised. The documents and literature content were used to form an interview guide. The primary data were collected through semi-structured in-depth interviews, which provide a balance between predefined and follow-up questions. According to Thagaard (2009) this is suitable to investigate the informant's perspectives. The informants were selected because they had been involved in the public procurement of EVs but were spread in terms of geographical location and type of activity. The sample, therefore, lent a strategic approach to our selection (Yin 2018). Questions were asked concerning the three primary areas of sustainability found in documents and literature (environmental, social, and economic), along with a wish to explore the understanding of sustainability and the participant’s perception of the effects of public procurement.

The interviews were conducted between January and May 2021. The informants had different roles in the procurement system and worked as advisers in state agencies, public procurement officers, public sector car suppliers, and end-users of government cars. Government agencies are state-controlled organisations that act independently to conduct the government's policy on procurement. The agencies serve as the government's expert body and develop guidelines based on laws and regulations. Public procurement officers are responsible for the procurement of goods and services that will help achieve the goals set by the government. Generally, their work entails finding suppliers through public procurement competitions where the goal is to strike a balance between quality and cost, where sustainability factors could be part of quality. The car supplier participates in these competitions to make cars available for the public entity. This level contains commercial-based stakeholders. The end-users are the ones who ultimately use the vehicles. The interviews lasted approximately one hour each and were conducted as digital video meetings due to covid-19. We stopped collecting data or interviewing when we reached a data saturation state (Guest et al. 2006). Table 2 shows an overview of the informants. The study will use the informants’ reference coding to present the results.

**Table 2:** Informants

Stakeholder	Role	Interview type	Reference
Government agency	Senior advisor	Digital	(A)
Government agency	Senior advisor	Digital	(B)
Government agency	Advisor	Digital	(C)
Municipality	Procurement officer	Digital	(D)
Municipality	Procurement officer	Digital	(E)
Health trust	Procurement officer	Digital	(F)
Car supplier	Car salesman	Digital	(G)
Car supplier	CEO	Digital	(H)
Car supplier	Manager	Digital	(I)
Inhabitant	End-user	Digital	(J)
Inhabitant	End-user	Digital	(K)
Inhabitant	End-user/patient	Digital	(L)

### Data analysis

**Fig. 2:**
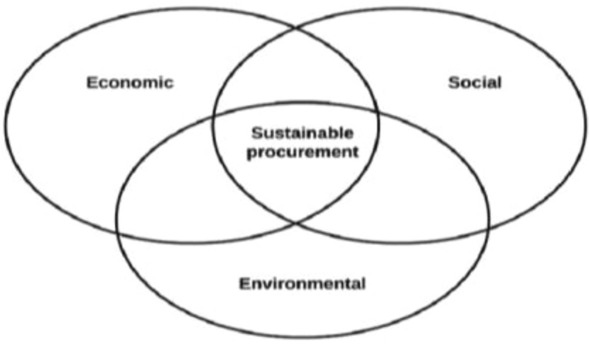
Triple bottom line of sustainability (Elkington 1997)

As preparation for analysis, the interviews were recorded and transcribed. The interviews were then classified using coding as the first step in the content analysis. To encode the data, we used open coding, gave keyword designations to the various elements respondents had provided, and then grouped these with similar answers (Yin 2018). Data were then categorised with different tags in relation to the themes (Sarker, Lau and Sahay 2000). By finding unifying headlines and grouping the initial tags, we developed new categories. Through repeated, systematic reviews of the dataset considering our categories and codes, we gradually developed a set of themes that represented the content of our dataset. The themes will be exemplified and presented in tables and quotes from the stakeholders. The study uses the accepted Triple Bottom Line approach (Elkington 1997) to organise the findings (see Fig. 2). The Triple Bottom Line is a framework that combines three different dimensions of sustainability: environmental, social, and economical.

This framework incorporates ecological and social measures demanding to assign appropriate means of measurement (Liute and De Giacomo 2021; Pedroso et al. 2021; Rogers and Ryan 2001; Slaper and Hall 2011). The study used public procurement decision-making as a management control activity as a theoretical lens to discuss the results. We wanted to understand better the stakeholder's perspectives associated with public procurement in the context of EVs. It was relevant to look closer at how the controllers, in this context government agencies and procurement officers, use data to manage and receive feedback in controlling the system considering the De Leeuw (1976) model.

## Results

If we first start looking at the concept of sustainability, we find a significant variation in the informants’ interpretation of the term. The government agencies emphasise the Triple Bottom Line approach from Elkington (1997). As stakeholder B said, “Simplistically speaking, sustainability is the environment, the society, and the economy, these three elements. It is a simplification of the 17 SDGs”. When we look at the sustainability concept from the procurement officers’ point of view, it is slightly more unclear. As stakeholder D said, “Sustainability includes the climate, the social perspective, and the circular economy. There is a lot in this concept. It means that we are doing something to better the livelihoods in our city”*.*

The car suppliers have their interpretation of the concept of sustainability. One talks about sustainability as an innovation opportunity, but the other two talk about it as an economic problem. As stakeholder G said, “I see sustainability as an opportunity for innovation. I will give you one example: I met with the home care services at a municipality yesterday; they have 100 cars. That is because, between 8:00 a.m. and 2:00 p.m., they need 100 cars. Between 2 p.m. and 10 p.m., they might need 70 cars. Furthermore, on weekends, they need 50. So, I challenge municipalities to buy vehicles to cover their needs in the middle range and have other solutions for the peak hours. This is a new solution that we have developed”. This is opposed to stakeholder I, who said, “If a tender is abnormally below the normal price, then there may be something wrong, it is not sustainable for car suppliers to provide a tender that is so low. And I guess I feel like we have done that, on the last tender”*.*

Thus, the concept of sustainability varies in interpretations. Governments and car suppliers live in slightly different realities. In the next section, we look closer at what they think about the environmental effects.

### Environmental effects

When it comes to the environmental effects of EVs, the opinions of the different stakeholders differ. In the interviews with the government agencies. Stakeholder A said, “What is the meaning of the term environmental is up to politics to define. The concept changes from time to time. However, right now, they are very concerned about zero-emission solutions. This is also reflected in the government’s climate plan for 2030. Our job as an agency is not to define political goals, but to take the political goals and turn them into action”.

The public procurement officers shared the vision of the government agencies to contribute to zero-emission cars with the increased number of EVs in their community. They had various explanations as to why. One said it was part of a political strategy to be at the forefront of reducing greenhouse emissions, and another said it was part of a plan to have a fossil-free vehicle fleet by 2023. However, all of them mostly talked about the environmental effects pertaining to the reduction of CO2 emissions. Stakeholder D explained, “The reason that this is an environmentally friendly project is that we get more electric cars, we get fewer fossil cars and less CO2 in our city centre”. None of the procurement officers brought up life cycle analysis or battery waste issues.

Car suppliers had different opinions. Two out of three did not agree with EVs having any environmental effects. As stakeholder G said, “I do not see any environmental effects of EVs. There are more factors to cars than just CO2. Taking the whole life cycle from production until disposal is the environmental math here. In 2007 everyone was going to drive diesel cars. And then suddenly, a few years later, we found out that there was something called particles. Then suddenly, it is not that environmentally friendly after all. When I hear about the environmental accounting of electric cars, I do not know if it is good overall. However, in terms of emissions, I am sure it is. However, I guess when the government has chosen to buy EVs, they have probably done an investigation to find out that this is wise”.

When the end-users talked about effects, they saw EVs as a way of ‘doing good’ if it worked in their daily routine. They liked the experience of driving an EV and felt that they contributed to the government's overall goals. Informant J expressed it this way: “I like to use my fossil car as little as possible, at least for work purposes, where there are opportunities for me to use electric cars. Moreover, it is an effortless choice, and there is no obstacle”*.* One interesting finding was that although most informants talked positively about the environmental effects of EVs, 7 out of the 12 did not own an EV themselves.

### Social effects

When it came to the social effects, the stakeholders were interested in different things. On the agency level, they saw the social side but mainly talked about sustainability's environmental side. Stakeholder A said, “There are several municipalities with more cars than population. There is a high suspicion that some of these cars are used very little. Then it is more efficient with a pooling system. When you talk about a pooling system, you start to take in the social perspective. However, I would say that the biggest push for us is to reduce greenhouse gas emissions related to transport. There is so much climate focus now”.

On the other hand, the procurement officers talked enthusiastically about the social effects. As procurement officer, stakeholder D said, “Access to cars has a social perspective. It makes it possible for those who do not have a car to be part of the community. The possibilities could include visiting the grave of a late spouse or allowing their children to join sports teams. Access to cars has a social side because we saw that children who did not have parents with a car fell out of organised life earlier than others”. The end-users in our material did not see the EVs having social effects. As stakeholder J said, “I do not see any social effects. Not in practice. I am so lucky that I have the alternative to use my own car”. For government agencies, the environmental effects were most important. The procurement officers cared about the social impacts, and the end-user was mainly concerned with the EV’s practical side.

### Economic effects

Stakeholders also had differing opinions when it came to the economic effects of EVs. Government agencies were primarily concerned with making administrative solutions for financing the shift to EVs. As stakeholder A said, “One of the ways for a municipality to finance EVs can be loans. It is a barrier to investing in advance; the municipal budget rules do not like that. Another way is to subsidise, but promoting that can sometimes be complicated because the mechanism is incompatible with public procurement. You need to show that the funding itself will trigger an environmentally friendly solution to get subsidies. The problem for municipalities is that they often must make a procurement competition first. And then they cut themselves off from getting subsidies. So, it is like a catch 22 situation”. When it comes to the procurement officers, they do not find the economic side of EVs that important. As stakeholder D said, “If you look specifically at electric cars, we have not put much emphasis on following up on the economic costs, because of the attitude towards it is that we are going to have zero-emission cars; It costs whatever it costs”. From the supplier side, they were more concerned with the economic effects. “What you are left with economically with each car is very small. EVs do not need, for example, oil. The procurement officers drive us hard, and I sometimes wonder if they want us to survive. So financially, it is not good”.

For government agencies, administrative solutions to cover political goals were most important. The procurement officers did not emphasise the cost. Some car suppliers worried about the economic impact, but others saw it as an opportunity for new business models. The end-users wanted solutions that worked their day-to-day life.

### Cultural effects

Cultural effects of sustainable procurement relate to whether there is a cultural shift in how stakeholders and society address economic, social, and environmental issues. Thus, the culture related to sustainability refers to people changing their consumption patterns and adapting to EVs. Procurement officer E explained it as follows: “There are many employees who have never used electric cars, who are now forced to use electric cars. And some then see that it works. I am sure that this has had ripple effects in this community, and from that, more people have gained experience with other types of cars”. The car suppliers look at the cultural aspect slightly different. As stakeholder, I said, “In the beginning, there were many who were sceptical, and there are still some who do not yet believe in electric cars. However, when you look at the number of electric cars sold in Norway, you notice that it has changed the entire car market in just a few years. Now the electric car is established in the minds of the entire staff. The attitude is that the electric car is here to stay, so we just have to deal with it”. The procurement officers emphasised that their change in public procurement practices enables a cultural shift towards sustainability through positive experiences with EVs, thereby influencing consumption patterns for their employees and the attitude of car suppliers.

### Innovation effects

Furthermore, we also find some innovation effects in our material. Government agencies put a strong emphasis on innovation. As it says in the white paper to the parliament, St. Meld. 22, 2019, “The Government wants the public sector as a customer to contribute to the use and development of new environmentally friendly technologies, products, and solutions. This is an important part of the policy for the green shift and for Norway to achieve our goals in the climate and environmental field. The public sector must adapt and solve its tasks in new ways”.

A stakeholder from government agencies (B) explains innovation as follows: “Innovation is not one thing, but several things. To make it happen, you must have clear political signals and support from the management; you need a person who does that little extra in the organisation, can handle procurement appropriately and get a good dialogue with the supplier market. However, simultaneously, they also need to look internally and work with the organisation to adopt the new solution they buy. If all these things are in place, then it can become an inspiring and good solution”. As we can see, sustainable innovation is an important goal for government agencies. The procurement officers are also aware that change requires innovation. However, innovation also comes with resistance from the rest of the organisation. As stakeholder F explained, “I must emphasise that innovation is a long road from the time we started the process internally until we have the environmental focus we have now. It has been going on for years. So, gaining acceptance for it internally has been, perhaps, the biggest obstacle”. Table 3 sums up the findings of RQ1 and shows the different stakeholders’ perspectives.

**Table 3:** Effects from the stakeholders’ perspective

Stakeholder/Effects	Environment	Social	Economic	Cultural/Innovation
Government agency	Wide scope of sustainability where environmental effects are defined as more than CO2	Social effects are important, but environmental effects matter more in the current political climate	Economic effects are discussed, but they are more concerned with environmental effects; one stakeholder considers this a prerequisite for the others	High emphasis on innovation
Procurement officers	The procurement of new cars becomes almost exclusively electric cars and focus on reducing CO2	Social effects are highlighted because the purchases give more user groups access to affordable car transport	Not so interested in economic effects; it is more an argument for change	High emphasis on cultural effects and ripple effects
Car suppliers	Different views on environmental effects, the life cycle of the car, and the total effect are discussed	See both positive and negative social effects—positive in terms of accessibility and negative in rural areas	Divided when it comes to economic factors, some see it as a loss, others as an opportunity	One sees this as an opportunity for innovation, but the two others are more reluctant to change; they all acknowledge that EVs have changed their culture
End-users	Enjoy the feeling of contributing to the environment when driving an EV	See the social aspect as an extra benefit	Thinks about the economic effect if it affects themselves	Some liked new opportunities, and others felt things should stay the same

The results show that the various stakeholders did not internalise the same elements of their calculations. They have different interests, ownership, backgrounds, and positions in the public procurement processes. Thus, there is an existence of various calculation practices rather than a uniform sustainability calculation process. The stakeholder’s view of effects is inconsistent, and they have local interpretations of which effects are most important. The government agencies present the ‘ideal version’ of public procurement; the procurement officers present how they do it ‘in practice’, and the suppliers and end-users talk about the consequences of the government officials’ decisions.

### Assessment of effects

According to the Cambridge Dictionary, assessment is about deciding the amount, value, quality, or importance of something. We found that stakeholders act as calculation agents, framing and externalising various procurement elements when constructing calculations. Both quantitative and qualitative components are included in the calculations. Externalities were not calculated and are therefore without value. The different actors do not internalise the same elements in their calculations. Thus, there are many calculation practices rather than a uniform sustainability calculation procedure. When we talk with the informants, the government agencies consider it ideal to base their decisions on feedback mechanisms in the form of data. Stakeholder A said, “We had a meeting with a company that was very concerned with value creation. The first question asked, who should we create value for? The second question is, what value should we create? The third question is, how should we measure value creation? Furthermore, the answer to the last question is often lost in what we do. Still, I think it is crucial”.

The public procurement officers do not have tools or systems to calculate the effects and find solutions that are ‘good enough’. As stakeholder D said, “I am sure that this has ripple effects in this community and see that more people have gained experience with other types of cars, but we do not measure it”. The car suppliers assess only the effects connected to their financial goals. As mentioned before, the supplier informants in our study differ in opinion. Some see EVs as an opportunity, and others see them as a threat. The end-users did not reflect on the assessment if the solutions worked in their daily routines.

In summary, it is the procurement officer's job to find the best solution to buy based on their interpretation of signals from the government agencies. However, they lack a feedback mechanism in the form of data to investigate and control if the goals are met. The procurement system has few organised data-driven channels for supplier or end-user input. There are no system or national guidelines for what should be included in sustainability assessment or which calculation methods should be used. Therefore, the different stakeholders do their own calculations in their evaluation on what to include and exclude. The government agencies' and procurement officers’ decisions could therefore lead to sustainability effects but also sub-optimal solutions for suppliers and end-users. To sum up the findings on RQ2, Table 4 shows how the different stakeholders perceive the assessment of the effects.

**Table 4:** Assessment of effects

Agency	Procurement officer	Car supplier	End-user
Express that the procurement officers should follow-up on the government’s ambitions It is ideal to have information and feedback in the form of data, but they are not there yet	Lack information systems and find solutions that are ‘good enough’ when calculating effects	Assess the effects connected to their financial goals but do not report data back to the procurement officers	Assess the effects for themselves but do not reflect on the assessment or feedback if the solutions work in their daily routines

## Discussion

This study first aimed to investigate how government agencies, procurement officers, car suppliers, and end-users perceive the effects of public procurement of cars. The findings show that different stakeholders operate with varying definitions of sustainability, and the sustainable aspects from Elkington (1997) are perceived in different ways in their assessment. In government agencies, environmental and quantitative effects, such as CO2 numbers, dominate over qualitative effects like user satisfaction. The public procurement officers and car suppliers emphasise the qualitative impact, and several stakeholders mention the social, innovation, and cultural elements. In sum, the results are in line with findings from Boiral et al. (2020), that sustainability is not a straightforward concept. The stakeholders do not internalise the same elements in their calculations, and there are many calculation practices rather than a uniform calculation process.

The second research question was related to how the stakeholders assess the effects of sustainable public procurement. When we look at our material in the light of De Leeuw’s control paradigm model (1976) and the preconditions for adequate control, the controllers, in this context the government agencies and public procurement officers, lack an objective and an evaluation mechanism. This is also because the controllers lack a controlled system model and information. Owing to the shortage of feedback mechanisms, the government officials in our material make decisions but do not have ways to evaluate whether their goals are met. The possibility of controlling actions to cope with variability is not being used because of a lack of feedback loops. Therefore, as illustrated in Fig. 2, controlling the procurement system is like one stakeholder said, “driving through a dense fog”, and the government decision-makers are left in the dark.

The stakeholders in our material act as calculation agents and frame and externalise various elements of sustainability when they make and construct calculations. In this sense, stakeholders have a range of different calculation practices rather than a unified understanding of a sustainability calculation. The procurement officers who are active early in the planning phase will, for example, make different calculations compared to the supplier stakeholders who are responsible for delivery. The controllers, in this case, are the government agencies and the public procurement officers. From the government agencies, sustainable procurement decisions are expected to demonstrate high levels of environmental value. Like one stakeholder said, “what is the meaning of the term environmental is up to politics to define. However, right now, they are very concerned about zero-emission solutions”. From the procurement officers’ point of view, social effects are as important. As one representative said, “Access to cars has a social perspective. It makes it possible for those who do not have a car to be part of the community”. This implied more driving which was not intended by the government's goals. From a practice and policy perspective achieving sustainable procurement will often involve balancing conflicting social and environmental values. Without common goals and understanding, it is difficult to achieve what De Leeuw (1976) calls an “evaluation mechanism to check whether the goals are met”.

The findings of this study offer valuable insights with implications for theory, practice, and policy, as discussed below. In terms of theory, the overall results support DeLeeuw’s (1976) model and the idea that public procurement decision-making can be seen as a management control activity where sustainability can be seen as an emergent property of the system (Lanhoso and Coelho 2020). Sustainability arises from the contributions made and conditions created in service of a shared reality. All the stakeholders’ decisions and calculations impact the system and affect the controller’s ability to control its system successfully. This confirmation is crucial because it affirms the relevance of this theory. Besides confirming the relevance of the management control theory, this study extends it by using it in a public procurement setting and showing that decision-making in sustainable public procurement should be seen as a non-linear process. The study indicates that there are many different calculation practices connected to the effects of public procurement. The effects are characterised by negotiations of reality by different stakeholders and therefore cannot be mandated from above. The government agencies and procurement staff make decisions but do not collect feedback from suppliers and end-users, and limited feedback loops exist. The finding supports the study by Michelsen and DeBoer (2009), which showed that problems with understanding effects could result from a lack of vertical integration across system levels, not merely from deficiencies at any one level alone. This is in line with findings from Moe (2006) and Sparrevik et al. (2018), which showed that various actors put different meanings into the concept of environment-friendly housing, and co-understanding was critical for success.

**Fig. 3:**
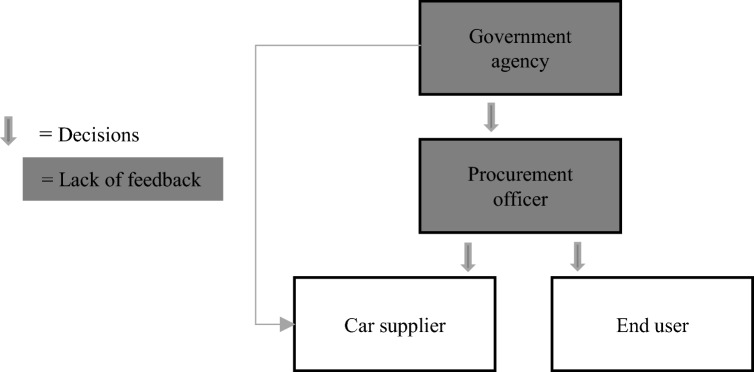
Model of the procurement system based on our findings

**Fig. 4:**
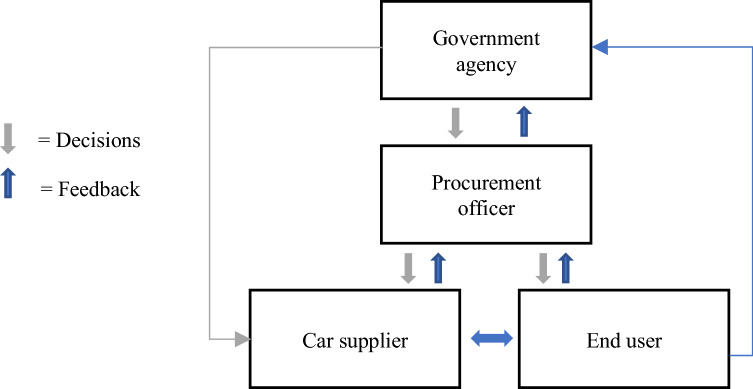
Model of control of public management of sustainability with feedback mechanisms for more informed decisions

The lack of vertical integration is caused, in part, by a lack of feedback mechanism between the different levels of the system. Stakeholders at one level, like government agencies, cannot see how their decisions interact with those made by actors at other levels. Like one of the representatives from the government agencies said, “how should we measure value creation? the answer to the question is often lost in what we do”. When we look at the stakeholders, CO2 is calculated, but only the informants from the car suppliers mentioned indicators related to life cycle analyses or battery waste. The Life Cycle Analysis was externalised in the calculations for the procurement officers. When we compare the lack of feedback loops in our findings with the model presented by De Leeuw, in an ideal setting, the controller’s ability to successfully control its system depends on vertical integration where the government agencies and procurement officers have information about the environment and the controlled system. To be in control of the system, a start would be to have an objective and evaluation mechanism and draw an ideal model of the procurement system. As illustrated in Fig. 3, government agencies and procurement officers would have better control with better information flow between the levels and probably make better decisions (Fig. 4).

If we look towards policy implication, the findings presented fall within the broader debate about digital transformation in the public sector (Mergel et al. 2019). There is a need to create processes and structures that facilitate feedback mechanisms for decisions. Elucidating how decision-making within public procurement can become data-driven, thus providing a foundation for improving the quality of the decisions, represents an example of how policies can be strengthened towards better feedback mechanisms within sustainable public procurement.

### Limitations

There are some limitations to this study. One of them is that the perceived effects can relate to a range of factors, such as organisational or political, and are often not directly related to public procurement itself. This is also seen in the results of our study. Another limitation could be in our strategic sample; by that, we mean the stakeholders selected as informants represented government entities that have paid attention to the procurement of EVs. This might not be representative of the larger population of public procurement entities. At the same time, from 2022, the public sector must buy EVs, and all public procurement entities will have to relate to these questions.

## Conclusion and further research

The Paris agreement (2015) and other sustainability initiatives e.g. (fit for 55, 2021) consider car transport a significant problem for the climate. EVs are considered part of the solution, and public procurement has been seen as a tool to push this forward. Despite the growing body of literature on sustainable public procurement, there is still a need for understanding the concepts and the calculation practices of effects. This paper seeks to fill a gap in the current literature by examining how various stakeholders perceive the effects. The study contributed with an awareness of missing links in the procurement system where there is a lack of feedback mechanism and shared understanding of effects. Public procurement is part of a complex system with various stakeholders. The problem is that we do not know the effects of public procurement decisions and how different stakeholders calculate the impact. According to De Leeuw’s (1976) control paradigm, the stakeholders need a model of the controlled system to evaluate and predict the effect of the decisions and actions. Therefore, the study visualised the current situation of the public procurement system and demonstrated a need for better vertical integration by using the De Leeuw model in a public procurement setting. The study intends to inspire the stakeholders to develop a practice to co-produce knowledge and use a data-driven feedback mechanism to contribute to more informed decisions within the public procurement context. For further research, there would be a need to expand the findings in a larger setting with quantitative methods to see if the perceived effects by our stakeholders are also represented in a larger sample. To better understand how knowledge is produced around sustainable public procurement, there would be interesting for further research to explore the co-creation process thoroughly, especially the cultural effects mentioned by several stakeholders. A better understanding of the perceived effects by various stakeholders in the procurement system could help government agencies to shape policy for better feedback mechanisms and find the way out of the fog.

## References

[CR1] Aasness MA, Odeck J (2015). The increase of electric vehicle usage in Norway—incentives and adverse effects. Eur Transp Res Rev.

[CR2] Aldenius M, Khan J (2017). Strategic use of green public procurement in the bus sector: challenges and opportunities. J Clean Prod.

[CR3] Alhola K, Ryding SO, Salmenperä H, Busch NJ (2019). Exploiting the potential of public procurement: opportunities for circular economy. J Ind Ecol.

[CR4] Alvesson, M., & Sköldberg, K. (2009). Reflexive Methodology: New Vistas for Qualitative Research (2nd ed.). London: Sage. ISBN 9781473964242

[CR5] Anthony, R., Govindarajan, V., (2007). Management Control Systems. 12th ed. McGraw-Hill, New York. ISBN-13978-0073100890

[CR6] Boiral O, Brotherton M-C, Talbot D (2020). Building trust in the fabric of sustainability ratings: an impression management perspective. J Clean Prod.

[CR7] Brammer S, Walker H (2011). Sustainable procurement in the public sector: an international comparative study. Int J Oper Prod.

[CR8] Braun, V., & Clarke, V. (2013). Successful qualitative research: A practical guide for beginners. London: Sage. ISBN 9781847875822

[CR9] Braun, V., & Clarke, V. (2021). The ebbs and flows of qualitative research: Time, change and the slow wheel of interpretation. Temporality in qualitative inquiry: Theories, methods, and practices. Routledge. ISBN 9780367538514

[CR10] Brynjolfsson, E., Hitt, L. M., & Kim, H. H. (2011). Strength in numbers: How does data-driven decision-making affect firm performance? SSRN1819486. 10.2139/ssrn.1819486

[CR11] Chen Y-C, Lee J (2018). Collaborative data networks for public service: governance, management, and performance. Public Manag Rev.

[CR12] Eccles RG, Ioannou I, Serafeim G (2014). The impact of corporate sustainability on organisational processes and performance. Manag Sci.

[CR13] Edler, J., Ruhland, S., Hafner, S., Rigby, J., Georghiou, L., Hommen, L., & Papadakou, M. (2005). Innovation and public procurement. Review of issues at stake. ISI Fraunhofer Institute Systems and Innovation Research, Karlsruhe. ISBN: 978 1 78347 188 1

[CR14] Edmondson AC, McManus SE (2007). Methodological fit in management field research. Acad Manag Rev.

[CR15] Eisenhardt KM (1989). Building theories from case study research. Acad Manage Rev.

[CR16] Elkington J (1997). Cannibals with forks: the triple bottom line of 21st century. Capstone Pub Oxford.

[CR17] Elkington, J., (2018). 25 years ago I coined the phrase “triple bottom line.” Here’s why it’s time to rethink it. Harv. Bus. Rev. 25. https://hbr.org/2018/06/25-years-ago-i-coined-the-phrase-triple-bottom-line-heres-why-im-giving-up-on-it . Accessed 22 Feb 2021

[CR68] European Union (2021) 'Fit for 55' delivering the EU's 2030 climate target on the way to climate neutrality. https://eur-lex.europa.eu/legal-content/EN/TXT/PDF/?uri=CELEX:52021DC0550&from=EN. Accessed 10 Nov 2021

[CR18] Fonseca LM, Domingues JP, Dima AM (2020). Mapping the sustainable development goals relationships. Sustainability.

[CR19] Gabriel Y (2018). Interpretation, reflexivity and imagination in qualitative research. Qualitative methodologies in organization studies.

[CR20] Glaser BG, Strauss AL (2017). The discovery of grounded theory: strategies for qualitative research.

[CR21] Gleeson-White, J., (2015). Six capitals, or can accountants save the planet? Rethinking capitalism for the twenty-first century. WW Norton &Company, New York. ISBN-13: 978-0393246674

[CR22] Grant BM, Giddings LS (2002). Making sense of methodologies: a paradigm framework for the novice researcher. Contemp Nurse.

[CR23] Guest G, Bunce A, Johnson L (2006). How many interviews are enough? An experiment with data saturation and variability. Field Methods.

[CR24] Ionescu I (2021). Corporate environmental performance, climate change mitigation, and green innovation behavior in sustainable finance. Econ manag financ mark.

[CR25] Ionescu I (2021). Leveraging green finance for low-carbon energy, sustainable economic development, and climate change mitigation during the covidpandemic. Rev contemp philos.

[CR26] Ionescu I (2021). Transitioning to a low-carbon economy: green financial behavior, climate change mitigation, and environmental energy sustainability. Geopolit hist internat relat.

[CR27] Keane J (2020). Can self-driving cars lead to sustainability? Autonomous smart sensors, perception and planning algorithms, and data processing efficiency. Contemp Read Law Soc Justice.

[CR28] Kemp R, Rotmans J, Elzen B, Geels FW, Green K (2004). Managing the transition to sustainable mobility. System innovation and the transition to sustainability: theory, evidence and policy.

[CR29] KPMG (2017) The time has come. The KPMG Survey of Corporate Responsibility Reporting. https://assets.kpmg/content/dam/kpmg/xx/pdf/2020/11/the-time-has-come.pdf. Accessed 22 Feb 2021

[CR30] Langseth M, Haddara M (2021) Exploring data analytics adoption in public procurement: the case of Norway. In: Langseth M, Similä JO (eds) Å kjøpe for Norge, Chap 9. Cappelen Damm Akademisk, Oslo, pp 223–256. 10.23865/noasp.128.ch9

[CR31] Lanhoso F, Coelho DA (2020). Emergence aiming innovation for sustainability. Syst Des Anal.

[CR32] De Leeuw A (1976) The control paradigm as an aid for understanding and designing organisations. TH Eindhoven., rapport, 36. https://pure.tue.nl/ws/files/4331695/30196.pdf. Accessed 22 Sept 2021

[CR33] Liute A, De Giacomo MR (2021). The environmental performance of UK-based B Corp companies: an analysis based on the triple bottom line approach. Bus Strateg Environ.

[CR34] Makri C, Neely A (2021). Grounded theory: a guide for exploratory studies in management research. Int J Qual Methods.

[CR35] May AYC, Hao GS, Carter S (2021). Intertwining corporate social responsibility, employee green behavior and environmental sustainability: the mediation effect of organizational trust and organizational identity. Econ Manag Financ Mark.

[CR36] Meld. St.13 (2020–2021) Klimaplan 2021–2030. Ministry of Climate and Environment. https://www.regjeringen.no/contentassets/a78ecf5ad2344fa5ae4a394412ef8975/nn-no/pdfs/stm202020210013000dddpdfs.pdf. Accessed 20 Jan 2021

[CR37] Meld. St.22 (2018–2019) Smartere innkjøp – effektive og profesjonelle offentlige anskaffelser. Ministry of Trade, Industry and Fisheries. https://www.regjeringen.no/contentassets/2d7006f67c374cbdab5d4d6ba7198ebd/no/pdfs/stm201820190022000dddpdfs.pdf. Accessed 10 Mar 2021

[CR38] Mergel I, Edelmann N, Haug N (2019). Defining digital transformation: results from expert interviews. Gov Inf Q.

[CR39] Michelsen O, de Boer L (2009). Green procurement in Norway; a survey of practices at the municipal and county level. J Environ Manage.

[CR41] Moe, H.T., (2006). Tro, håp og hybrid ventilasjon-mål på miljøvennlighet i bygninger. PhD thesis. NTNU Open. 10.18261/issn.2535-2512-2019-05-01

[CR42] Mulligan K (2021). Computationally networked urbanism and advanced sustainability analytics in internet of things-enabled smart city governance. Geopolit Hist Int Relat.

[CR43] NEA (2020) Klimakur 2030. https://www.miljodirektoratet.no/globalassets/publikasjoner/m1625/m1625.pdf. Accessed 22 Feb 2022

[CR44] Neri A, Cagno E, Lepri M, Trianni A (2021). A triple bottom line balanced set of key performance indicators to measure the sustainability performance of industrial supply chains. Sustain Prod Consum.

[CR45] Nilsen HR (2020). Staying within planetary boundaries as a premise for sustainability: on the responsibility to address counteracting sustainable development goals. Nord J Appl Ethics.

[CR46] Norwegian Agency for Public and Financial Management (2020) Norwegian maturity survey for public procurement. https://anskaffelser.no/sites/default/files/modenhetsrapport_v4_2021.pdf. Accessed 10 Mar 2021

[CR47] Nykvist B, Nilsson M (2015). The EV paradox–A multilevel study of why Stockholm is not a leader in electric vehicles. Environ Innov Soc Transit.

[CR48] OECD-MAPS, Methodology for Assessing Procurement Systems (2020) The assessment report, Sustainable Public Procurement in Norway. https://www.mapsinitiative.org/assessments/MAPS%20Norway%20SPP.pdf. Accessed 10 Mar 2021

[CR100] OFV (2022) Record year for new car registrations. https://ofv.no/produktinfo/2021-was-the-record-year-for-new-car-registrations. Accessed 4 Jan 2022

[CR49] Palm J, Backman F (2017). Public procurement of electric vehicles as a way to support a market: examples from Sweden. Int J Electr Hybrid Veh.

[CR50] Paris agreement (2015) Report of the conference of the parties to the United Nations Framework Convention on climate change. https://unfccc.int/sites/default/files/english_paris_agreement.pdf. Accessed 10 Mar 2021

[CR51] Patrucco AS, Luzzini D, Ronchi S (2017). Research perspectives on public procurement: content analysis of 14 years of publications in the journal of public procurement. J Public Procure.

[CR52] Pedroso CB, Tate WL, da Silva AL, Carpinetti LCR (2021). Supplier development adoption: a conceptual model for triple bottom line (TBL) outcomes. J Clean Prod.

[CR53] Porter, M. E., & Kramer, M. R, (2011). Creating Shared Value: Harvard Business Review. From the Magazine (January–February 2011)

[CR54] Rogers M, Ryan R (2001). The triple bottom line for sustainable community development. Local Environ.

[CR55] Rolfstam, M. (2015). Measuring effects of public procurement of innovation. In XIX IRSPM Conference

[CR56] Sarker S, Lau F, Sahay S (2000). Using an adapted grounded theory approach for inductive theory building about virtual team development. ACM SIGMIS Database.

[CR57] Schaltegger S, Burritt RL (2010). Sustainability accounting for companies: catchphrase or decision support for business leaders?. J World Bus.

[CR58] Shepherd S, Bonsall P, Harrison G (2012). Factors affecting future demand for electric vehicles: a model based study. Transp Policy.

[CR59] Singh GG (2018). A rapid assessment of co-benefits and trade-offs among sustainable development goals. Mar Policy.

[CR60] Slaper TF, Hall TJ (2011) The triple bottom line: what is it and how does it work. Indiana business review 86(1):4–8. http://www.ibrc.indiana.edu/ibr/2011/spring/article2.html. Accessed 10 Mar 2021

[CR61] Sönnichsen SD, Clement J (2020). Review of green and sustainable public procurement: towards circular public procurement. J Clean Prod.

[CR62] Sparrevik M, Wangen HF, Fet AM, De Boer L (2018). Green public procurement–A case study of an innovative building project in Norway. J Clean Prod.

[CR63] Suddaby R (2006). From the editors: what grounded theory is not. Acad Manag J.

[CR64] Thagaard, T., (2009). Systematikk og innlevelse: en innføring i kvalitativ metode (Vol. 3). Bergen, Fagbokforlaget. ISBN 9788245023985

[CR65] Thai KV (2001). Public procurement re-examined. J Public Procure.

[CR66] Tran M, Banister D, Bishop JD, McCulloch MD (2013). Simulating early adoption of alternative fuel vehicles for sustainability. Technol Forecast Soc Change.

[CR69] United Nations, (2015), Transforming our World: The 2030 Agenda for Sustainable Development https://sdgs.un.org/sites/default/files/publications/21252030%20Agenda%20for%20Sustainable%20Development%20web.pdf

[CR70] Van Thiel S, Leeuw FL (2002). The performance paradox in the public sector. Public Perform Manag Rev.

[CR71] Van Rijnsoever FJ, Hagen P, Willems M (2013). Preferences for alternative fuel vehicles by Dutch local governments. Transp Res d Trans Environ.

[CR72] Vergragt PJ, Brown HS (2007). Sustainable mobility: from technological innovation to societal learning. J Clean Prod.

[CR73] Villareal A (2011). The social construction of the market for electric cars in France: politics coming to the aid of economics. Int J Automot Technol Manage.

[CR74] VNR ( 2021) Voluntary National Review Norway. Norwegian Ministry of Local Government and Modernisation. https://sustainabledevelopment.un.org/content/documents/28233Voluntary_National_Review_2021_Norway.pdf. Accessed 4 Oct 2021

[CR75] Weber J (2011) Drivers of successful controllership: activities, people, and connecting with management. Business Expert Press, New York. ISBN-13: 978-1606491041

[CR76] Whitmarsh L, Köhler J (2010). Climate change and cars in the EU: the roles of auto firms, consumers, and policy in responding to global environmental change. Cambridge J Reg Econ.

[CR77] Ydersbond IM (2018) A green dream: municipal cars driving on electricity. ISBN-13: 978-82-480-2173-5

[CR78] Yin RK (2018) Case study research and applications. Sage, Los Angeles. ISBN: 9781506336169

